# From Reified Identity to Relational Ipseity: Hermeneutic Phenomenology, Language Pragmatics, and Contemplative Practices of Self-Deconstruction

**DOI:** 10.62641/aep.v54i4.2217

**Published:** 2026-08-15

**Authors:** Roberto Aristegui Lagos, Javier García-Campayo, Paulina Lamas-Morales

**Affiliations:** ^1^Department of Psychiatry and Mental Health East, University of Chile, 750000 Santiago, Chile; ^2^Department of Medical Humanities and Family Medicine, University of Valparaíso, 2340000 Valparaíso, Chile; ^3^Department of Medicine, Psychiatry and Dermatology, University of Zaragoza, 50009 Zaragoza, Spain; ^4^Department of Psychology and Sociology, University of Zaragoza, 50009 Zaragoza, Spain

**Keywords:** ipseity, self-deconstruction, hermeneutic phenomenology, mindfulness, non-duality, clinical implications

## Abstract

This article presents a theoretical and conceptual analysis of self-deconstruction in contemporary contemplative and clinical practices. It argues that psychological suffering is not primarily related to the existence of the self as such, but to its ontological and linguistic reification as a fixed identity, corresponding to idem-identity and to what Heidegger described as an ontology of presence (Vorhandenheit). Drawing on hermeneutic phenomenology, the self is reconceptualized as ipseity (selfhood): a relational, temporal, and performative mode of being-in-the-world. From this perspective, self-deconstruction does not imply the elimination of identity but a transformation in the mode of being of the self. This framework integrates three axes: (a) Heidegger’s analytics of being-in-the-world and the distinction between sameness and ipseity; (b) a pragmatic distinction between indirect and direct reference in language, allowing experiential and clinical transformation without representational reductionism; and (c) a dialogue with non-dual contemplative traditions and contemporary clinical approaches, particularly mindfulness-based and third-wave therapies. The article proposes a translational architecture linking ontological analysis, phenomenology of language, and clinical practice, and shows how non-duality can be understood in a non-nihilistic way when grounded in an ontology of ipseity. Clinical implications for psychotherapy, mindfulness-based interventions, and models of self-related suffering are discussed, including connections to Acceptance and Commitment Therapy, cognitive defusion, and decentering, as well as boundary conditions and safeguards for contemplative practices.

## Introduction

Contemporary psychology has largely inherited a modern conception of the self in which the ego is understood as a relatively stable and identifiable entity open to description and intervention. While this approach has enabled important technical developments, it has also contributed to a systematic reification of identity by treating the self as an object available for observation and evaluation. This reification is included in what Heidegger referred to as an ontology of presence, where reality appears as present-at-hand (Vorhandenheit, literally “being available before one,” as opposed to the practical involvement of everyday engagement), separate from its practical and relational aspects [1]. Different phenomenological, pragmatic, and contemplative currents have questioned this implicit ontology, showing that psychological suffering is not adequately explained by the mere presence of dysfunctional mental contents but by rigid modes of self-reference and by the fixing of identity that impoverishes experience.

This article advances three interrelated claims. First, it argues that the deconstruction of the self does not involve its elimination but a transformation in its mode of being, from reified sameness (idem-identity) to relational ipseity (selfhood), which can only be understood when the self is conceived within a pragmatics of non-representational language and with the recognition that indeterminacy is an existential condition [2]. Second, if the suffering associated with self-related psychopathology arises primarily from the reification of identity rather than from the content of specific cognitions, then clinical interventions that loosen this reification—such as defusion, decentering, and contemplative self-inquiry—should produce change by transforming the mode of self-reference rather than by modifying the propositional content of beliefs. Third, if non-duality is understood not as a metaphysical thesis about the non-existence of the self but as a lived modality of being-in-the-world grounded in ipseity, then contemplative practices oriented toward non-dual experience can be integrated into clinical frameworks without nihilism or cognitive reductionism.

The notion of deconstruction of the self has gained growing importance in both philosophical and clinical reasoning. Nevertheless, this notion tends to oscillate between eliminativist interpretations—which understand the “non-self” as an ontological negation—and psychologized readings that reduce it to a cognitive technique. This article proposes that this ambiguity is due to insufficient ontological and semantic clarity regarding the self [2]. In recent years, contemplative science has begun to examine these questions within a broader empirical and theoretical framework. Attentional, constructive, and deconstructive families of meditation practices have been proposed as different pathways through which contemplative training may reshape the sense of self and the structure of experience [3]. Contemporary contemplative literature has also explored practices oriented toward non-duality and emptiness (the experiential recognition that the self lacks a fixed, independent essence) that investigate the constructed character of the self [4, 5, 6].

The text is presented as a theoretical and conceptual article whose contribution to psychology consists of providing a non-reductionist framework for understanding non-duality, mindfulness, and third-generation practices while avoiding nihilism and cognitive individualism. The article follows a translational architecture organized across three levels—ontological analysis, phenomenology of language, and clinical practice—each grounded in specific literatures and connected by explicit transitional bridges.

## Methodology: Hermeneutic-Phenomenological Conceptual Analysis

This article employs a hermeneutic-phenomenological method of conceptual analysis [1, 7] directed at the ontological and semantic presuppositions underlying contemporary accounts of self-deconstruction. The approach does not aim to produce empirical predictions but to clarify the conceptual conditions under which clinical and contemplative phenomena can be coherently understood.

The analysis is organized along three integrative axes. The first axis draws on Heidegger’s analytic of being-in-the-world [1] and Ricoeur’s distinction between idem-identity and ipse-identity [7] to articulate the ontological shift from a reified to a relational understanding of the self. The second axis integrates the pragmatics of language—Wittgenstein’s language-games [8], Gendlin’s direct reference and felt sense [9], and speech act theory [10, 11]—to show how language not only describes but constitutes modes of self-reference. The third axis bridges these philosophical analyses to clinical and contemplative practices, particularly mindfulness-based interventions, Acceptance and Commitment Therapy (ACT) [12], and deconstructive meditation [3, 13].

Source selection follows the hermeneutic principle of dialogical engagement rather than systematic exhaustive retrieval. Primary sources include the canonical texts of hermeneutic phenomenology (Heidegger [1], Ricoeur [7], Dreyfus [14]), language pragmatics (Wittgenstein [8], Austin [10], Gendlin [9]), enactive cognition (Varela *et al*. [15]), and contemporary contemplative science (Dahl *et al*. [3], García-Campayo [4], Van Gordon [5]). Clinical literature on ACT, defusion, and decentering is drawn from representative meta-analyses and theoretical syntheses rather than exhaustive systematic review.

The scope of the article is deliberately limited in three respects: (a) it addresses the ontological and semantic dimensions of self-deconstruction rather than its neural correlates, although neuroscientific findings are cited where they illuminate the argument; (b) it does not propose testable hypotheses but conceptual if-then claims that specify the conditions under which clinical phenomena become intelligible; and (c) it does not claim to represent all contemplative traditions but draws primarily on practices that have entered the clinical literature.

## Reification of the Self and Sameness (Level 1: Ontological Analysis)

The central problem addressed in this article is not the existence of the self but its reification as a fixed identity [13, 16]. When the self is understood through rigid descriptive categories, it is fixed as sameness (idem-identity, Ricoeur’s term for the “what” of identity that persists as a set of stable characteristics): something that can be defined, classified, and explained. This determination transforms the self into an object and separates it from its temporal, relational, and practical nature [7]. Constative language (language that describes or states facts) reinforces this ontology by treating identity as a set of stable predicates. From the Heideggerian [1] perspective, this understanding corresponds to an ontologization derived from human existence (Dasein) as present-at-hand, forgetting that the original mode of being of human existence is being involved in practices, ready-to-hand (Zuhandenheit, the mode of practical engagement), in a shared world.

This reification has direct clinical significance. Rumination, a transdiagnostic process linked to depression, anxiety, and other forms of psychopathology, can be understood as a form of compulsive constative self-reference in which the person repeatedly describes and evaluates themselves through fixed predicates (“I am defective,” “I always fail”) [17]. The suffering is not produced by the content of these descriptions alone but by the rigid mode of self-reference that treats the self as a determinable object. This ontological insight provides a bridge from philosophical analysis to the clinical phenomena that third-wave therapies seek to address.

## Ipseity and Hermeneutic Phenomenology

Heidegger [1] dismantles the subject-object model by showing that human existence is not primarily a consciousness that represents the world but being-in-the-world. The “self” does not designate a substance but a mode of being constituted by facticity, temporality, and project. Ipseity (from Latin ipse, “self” or “itself”) gives a name to this insubstantial dimension of the self: a who that unfolds over time, not a what that can be fixed. It is important to distinguish this use of ipseity from its clinical-phenomenological use in the schizophrenia literature, where Sass and Parnas [18] employ the term to designate the minimal or pre-reflective sense of self that is disturbed in schizophrenia-spectrum disorders. While both usages share a phenomenological lineage, the present article uses ipseity in the broader Heideggerian-Ricoeurian sense to denote the relational and temporal selfhood that is obscured by reification, rather than the minimal self-affection that is specifically disrupted in psychotic experience. The deconstruction of the self does not imply the elimination of the self but a transformation in the mode of selfhood [2, 13].

Deconstruction of the self, therefore, involves freeing ipseity from its capture by sameness. This shift is not merely theoretical but existential, and it is manifested through a transformation of the means by which the self is expressed linguistically and practically. This transformation marks the transition from the ontological level of analysis to the phenomenology of language.

## Language, Pragmatics, and Direct Reference (Level 2: Phenomenology of Language)

This shift requires letting go of the representational conception of language. From a pragmatic perspective inspired by Wittgenstein, meaning is not a correspondence between words and objects but a situated use in language-games (rule-governed forms of life in which meaning arises from practical activity) [8]. Within this framework, it is crucial to distinguish between indirect reference, which tends to objectivize experience, and direct reference, which allows meaning to emerge from the lived experience without being objectified [9]. Speech act theory [10, 11] explains that language not only describes but carries out actions: it configures modes of being. This performative turn allows us to understand identity not as a described object but as a way of responding and behaving in relational contexts [19].

From a clinical perspective, what contemporary third-wave therapies call “cognitive defusion” [12] and “decentering” [20] can be understood phenomenologically as precisely this shift from indirect to direct reference. In defusion, the client learns to relate to thoughts as transient linguistic events rather than as descriptions of reality—a shift from constative to performative language use. Gendlin’s concept of the “felt sense” [9] provides the experiential counterpart to Wittgenstein’s pragmatic theory: meaning is not grasped through conceptual analysis but through attending to the bodily sense of a situation, a pre-conceptual knowing that cannot be fully captured by constative language. In contemplative contexts, linguistic instructions often function less as descriptions of mental states than as orienting gestures—what Heidegger called “formal indications”—that guide experiential inquiry without defining in advance what will be disclosed [1].

This performative and non-representational use of language combines with enactive approaches that conceive of meaning as emerging from co-participation by body, world, and language, particularly in developments that integrate neurophenomenology and language theory [15, 21]. An analogous convergence between phenomenology and contemplative traditions is explored in Nishitani’s philosophy of emptiness [22], which resonates with the non-representational stance adopted here. The relation between linguistic instruction and experiential understanding is not determined by a single correspondence but emerges through situated interpretation, a situation reminiscent of what Quine [23] described as the indeterminacy of radical translation.

## Indeterminacy, Anxiety, and the Limits of Self-Reference

The abandonment of representationalism, expressed in Rorty’s pragmatic turn [24] and grounded in Wittgenstein’s language-games [8], Quine’s confirmation holism and underdetermination [23], and Sellars’s nominalism [25], has a direct consequence for self-reference. When applied to the self, the thesis of indeterminacy in radical translation leads to a double underdetermination: both the reference and the speaker’s mode of being are indeterminate. The self does not appear as an object but as a situated linguistic use, dependent on language-games and specific contexts [26].

Treating the “non-self” as an ontological thesis is, therefore, a categorical error. The indeterminacy of the self is not equivalent to the negation of existence but to the absence of a final objectifiable fundament. This absence has often been experienced and thematized as anxiety. According to Heidegger [1], however, Angst (anxiety in its existential, not merely clinical, sense) is not a pathology but a privileged access to the existential dimension of existence, when the securities of the They (das Man, the anonymous social conformity of everyday life) are suspended, and the horizon of the potentiality-for-being opens.

Clinically, this existential anxiety is relevant because rigid self-reification can be understood as a defense against indeterminacy—a refusal to tolerate the groundlessness that ipseity entails. From this perspective, the “psychological inflexibility” that ACT identifies as the core of psychopathology [12] is an ontological inflexibility: the insistence on maintaining a fixed self-description when the mode of being of the self is fundamentally open. The transition from ontological and linguistic analysis to clinical practice requires understanding how contemplative and therapeutic techniques facilitate this tolerance of indeterminacy.

## Contemplative Practice and Clinical Dereification (Level 3: Clinical Practice)

From a phenomenological perspective, contemplative instructions can be interpreted as formal indications in the sense of Heidegger. Rather than defining experiential states, they orient attention toward phenomena that can only be disclosed through practice. Attentional practices guide attention toward the field of experience, constructive practices reshape the affective relation to that field, and deconstructive practices investigate the constitution of the self within experience [3]. These distinctions should not be understood as a rigid taxonomy but as overlapping families of practices, reminiscent of Wittgenstein’s notion of family resemblance among language-games.

In the field of mindfulness, the notion of reperceiving has been described as a central mechanism of change [20]. However, without combining it with direct, embodied experience, reperceiving may be reduced to the metacognitive level. At this point, we see the importance of enactive and non-deliberative ethics proposed by Varela [15], in which ethical action emerges from an embodied sensitivity rather than from the application of normative principles. From the relational perspective, mindfulness can be understood as an intersubjective field in which the performativity of language and direct reference to experience play a key role in the dereification of the self, rather than individual attentional practice alone [27]. While this relational framework is theoretically grounded, its specific clinical mechanisms await empirical investigation beyond the preliminary evidence available for relational mindfulness approaches.

### Clinical Vignette: From Constative Identity to Performative Ipseity

The following composite clinical vignette, drawn from common patterns in contemplative-therapeutic practice, illustrates how this experiential reorientation unfolds. During a contemplative exercise in a group therapy session, a participant reports the persistent thought “I am a bad father.” Initially, the statement functions as a constative speech act—a fixed description of identity that the participant takes as simply true. The therapist does not challenge the content of the statement. Instead, she invites the participant to attend more closely to the unfolding experience: “Can you notice what happens in your body as you say those words? Where do you feel it? What is the tone of that voice?”

As the participant stays with these elements—the chest tightness, the heaviness, the self-critical tone—the statement begins to lose its character as a settled description. The therapist then introduces a performative reframe: “Try saying it again, but this time, notice that you are the one saying it—that these words are appearing in your experience right now.” The participant repeats the phrase, and something shifts: the sentence “I am a bad father” gradually appears not as a definitive identity but as a transient interpretation arising within experience. The participant reports: “It’s still there, but it feels more like… a thought that comes. Not the whole truth of who I am.”

In the terms of this article, what has occurred is a shift from indirect reference (treating the thought as a description of a fixed self-object) to direct reference (attending to the thought as it arises within the lived field of experience). The constative “I am a bad father” has not been replaced by another constative (“I am a good father”) but has been disclosed as a performative event—a language act that constitutes a mode of relating rather than a settled fact. Observable markers of dereification include: the shift from identification with the content to observation of its arising, the emergence of somatic awareness alongside the propositional content, and the loosening of the certainty that accompanies reified self-descriptions. This is the sameness-to-ipseity transformation at work in clinical practice.

### Integration With Existing Clinical Models

The framework developed in this article provides a philosophical grounding for mechanisms already employed in third-wave clinical approaches. In ACT, the process of cognitive defusion—learning to see thoughts as thoughts rather than as literal truths—corresponds to the shift from constative to performative language use described above. The ACT concept of “self-as-context” [12], in which the self is experienced as the perspective from which thoughts and feelings are observed rather than as their content, is a clinical approximation of ipseity: a relational, temporal who rather than a fixed what. Similarly, the decentering described in mindfulness-based cognitive therapy [20, 28]—the capacity to observe one’s thoughts and feelings as transient mental events rather than as reflections of reality—enacts the movement from indirect to direct reference.

This philosophical grounding matters clinically because it addresses the question of why these techniques work. If defusion, decentering, and self-as-context produce therapeutic change, it is not merely because they introduce a “cognitive distance” from negative thoughts but because they transform the ontological mode in which the self relates to its own experience: from reified presence-at-hand to engaged, relational ipseity. Neuroimaging evidence supports this interpretation: mindfulness training has been associated with reduced activity in default-mode network regions linked to self-referential narrative processing [29] and with a shift from narrative to experiential modes of self-reference [30], consistent with the shift from idem-identity to ipseity proposed here.

## Clinical Implications: Mechanisms, Techniques, and Boundary Conditions

The translational architecture developed in this article—from ontological analysis through phenomenology of language to clinical practice—yields several implications for psychotherapy and contemplative interventions.

### Mechanism of Change

The central therapeutic mechanism proposed is not the elimination of negative self-referential content but the transformation of the mode of self-reference from reified sameness to relational ipseity. This transformation involves three interrelated shifts: (a) from constative to performative self-language, (b) from indirect to direct reference in experiential awareness, and (c) from rigid identification with self-descriptions to the capacity to inhabit the indeterminacy of ipseity. These shifts align with the “psychological flexibility” model of ACT [12] but provide it with an ontological grounding that avoids reducing the therapeutic process to a set of cognitive techniques.

### Clinical Techniques

The framework supports a range of existing clinical techniques and suggests principles for their integration:

• Experiential exercises that invite attention to the embodied, situated arising of self-referential thoughts (as illustrated in the clinical vignette above), moving from propositional content to the felt sense of experience [9].

• Language practices that explicitly shift from constative to performative speech—for instance, reformulating “I am anxious” as “anxiety is arising in my experience”—not as a cognitive trick but as a genuine reorientation of the mode of self-reference.

• Relational mindfulness practices [27] in which the intersubjective field supports the loosening of rigid self-identification through shared attention and performative dialogue.

• Deconstructive meditation practices [3, 13] that investigate the constructed character of the self within experience, guided by formal indications rather than doctrinal assertions about the self.

### Empirical Landscape

The evidence base for these practices is uneven and requires honest acknowledgment. Meta-analytic evidence supports the efficacy of mindfulness-based therapies for a range of psychological conditions, with moderate to large effect sizes for anxiety, depression, and stress [31]. Studies on ACT demonstrate that psychological flexibility mediates therapeutic outcomes across multiple disorders [12]. However, the specific hypothesis that dereification of the self—as distinct from other mechanisms of mindfulness and ACT—constitutes an independent therapeutic pathway remains largely untested. Emerging empirical work on deconstructive meditation [32, 33] and self-inquiry practices suggests that these approaches may produce distinctive psychological effects, but the evidence base is still thin, and well-controlled trials with adequate samples are needed. The conceptual framework proposed here should be understood as providing conditions for generating testable hypotheses rather than as claiming established empirical support.

### Bridge to ACT, Defusion, and Decentering

The ipseity framework explains not only that defusion and decentering work but why: they succeed to the extent that they effect a genuine ontological shift in the mode of self-reference rather than merely introducing cognitive distance. This suggests that clinical protocols could be enriched by attending explicitly to the quality of self-reference (constative vs. performative, indirect vs. direct) rather than only to the content of cognitions or the degree of detachment from them.

### Boundary Conditions and Safeguards

Not all individuals may benefit equally from practices that loosen the reification of identity, and some may be harmed. Research on the adverse effects of meditation has identified a significant prevalence of challenging experiences, including anxiety, depersonalization, and destabilization of the sense of self [34, 35, 36]. These findings underscore that self-deconstruction practices should be understood as powerful interventions requiring appropriate clinical context, not as universally benign wellness techniques. Individuals with fragile or unstable self-structures—including those with psychotic vulnerability, severe dissociative tendencies, or acute trauma—may experience deconstructive practices as threatening rather than liberating. The distinction between ipseity and the minimal self-disturbance described in the schizophrenia literature [18] is clinically important here: contemplative dereification aims to loosen the reification of identity, not to further disturb a self-presence that is already fragile. Clinical implementation therefore requires careful assessment, graduated introduction of deconstructive elements, and the availability of stabilizing attentional and constructive practices [3] as scaffolding.

## Development of Contemplative Competence

This understanding of self-deconstruction as the transformation from reified sameness to relational ipseity is particularly relevant for understanding the progressive development of contemplative competence. This transformation in the relation to the self can be understood as a gradual development of practical competence, similar to the progression described by Dreyfus [14] in his interpretation of Heideggerian modes of being-in-the-world. Table 1 presents a developmental framework mapping stages of contemplative learning to Heideggerian modes of being and corresponding clinical observations. The progression is not strictly linear; practitioners may move between stages depending on practice context and life circumstances, and competent practitioners may revisit earlier stages when encountering new practices or unfamiliar experiential territory.

**Table 1.** **Development of contemplative competence and modes of being-in-the-world**.

Stage of learning	Heideggerian mode of being	Relation to the self	Clinical/contemplative example
Conceptual understanding	Purely present-at-hand	Reified identity: self understood through fixed categories	Client describes self in rigid terms: “I am an anxious person.” Meditator reads about non-self intellectually.
Beginner practitioner	Present-at-hand	Observed or conceptual self: attention directed at self as object	Client notices thoughts during mindfulness exercise but still identifies with content. “I’m watching my anxiety.”
Competent practitioner	Unready-to-hand	Destabilization of fixed identity: familiar self-categories feel uncertain	Client reports: “I’m not sure who I am without my worry.” Meditator experiences anxiety as self-descriptions loosen.
Advanced practitioner	Ready-to-hand	Relational ipseity: self as open, temporal, situated orientation	Client responds flexibly to self-referential thoughts without being captured. Meditator rests in awareness without needing to fix experience.

Note: Adapted from Heidegger [1] (modes of being-in-the-world) and Dreyfus [14] (skill acquisition). Progression is non-linear; practitioners may move between stages.

## Limitations

The present framework is subject to important limitations that should be acknowledged. First, from a neuroscientific-eliminativist perspective, Metzinger [37] argues that the self is a representational construct of the brain—a “self-model”—and that no entity corresponding to the self exists in the world. If Metzinger is correct that the phenomenal self is entirely constituted by subpersonal neural processes, the Heideggerian recourse to ipseity as a genuine mode of being may be philosophically unmotivated. The present article responds that the hermeneutic-phenomenological approach does not make claims about the neural constitution of selfhood but operates at a different level of description—the level of meaning and lived experience—that is not reducible to, though it may be constrained by, neuroscientific accounts. Second, an empiricist objection holds that a framework generating no falsifiable predictions has limited scientific value. This concern is legitimate, and the article does not claim to substitute for empirical investigation. Rather, it aims to provide conceptual clarity about what is being studied—the mode of self-reference rather than its propositional content—thereby generating more precise empirical questions than those currently framing the field. Third, the contemplative traditions engaged here—primarily Buddhist-influenced mindfulness and non-dual practices—represent a selective cultural sampling. Indigenous, Islamic, Christian contemplative, and other non-Western traditions offer alternative accounts of self-transformation that may complement or challenge the present framework, and future work should broaden the cultural scope of this inquiry.

## Crosswalk-Glossary: Philosophical and Clinical Terms

**Supplementary Table 1** provides a crosswalk between the philosophical concepts used in this article and their approximate clinical and contemplative equivalents, intended to facilitate dialogue across disciplinary traditions.

## Conclusions

The central argument of this article can now be stated in light of the preceding analysis. Self-deconstruction does not involve the elimination of the self but a transformation in its mode of being—from reified sameness to relational ipseity. This transformation is not merely theoretical: it is enacted through a shift in language use (from constative to performative self-reference), disclosed through a tolerance of the indeterminacy that ipseity entails, and cultivated through contemplative and clinical practices that loosen rigid self-identification without dismantling the capacity for self-presence.

The three conceptual claims advanced in the introduction can be assessed against the analysis. First, the distinction between idem-identity and ipseity provides the ontological resources to understand self-deconstruction as transformation rather than elimination, clarifying a persistent ambiguity in both philosophical and clinical discourse. Second, the phenomenological analysis of language—particularly the distinction between constative and performative speech, and between indirect and direct reference—illuminates why clinical interventions such as defusion and decentering are effective: they transform the mode of self-reference rather than merely modifying cognitive content. Third, the grounding of non-duality in an ontology of ipseity makes it possible to integrate contemplative practices into clinical frameworks without nihilism, reductionism, or the elimination of personal agency. Taken together, these findings support a shift from understanding the self as a fixed entity to approaching it as a relational and dynamically constituted mode of being.

## Availability of Data and Materials

Not applicable.

## Supplementary Material

Supplementary material associated with this article can be found, in the online version, at https://doi.org/10.62641/aep.v54i4.2217.
