# Supplementary material for: From Reified Identity to Relational Ipseity: Hermeneutic Phenomenology, Language Pragmatics, and Contemplative Practices of Self-Deconstruction
Source: Actas Esp Psiquiatr. 2026 Aug 15;54(4):1304–12. doi: 10.62641/aep.v54i4.2217 (PMC13571181; doi:10.62641/aep.v54i4.2217)
Supplement: Supplementary file 1 [file ActEsp-54-4-1304-1312-s1.zip › Supplementary_Table_1.docx]

**Supplementary Table S1. Crosswalk glossary of philosophical and clinical terms**

| Philosophical term | Clinical/contemplative equivalent | Brief definition |
| --- | --- | --- |
| Reification / presence-at-hand (*Vorhandenheit*) | Cognitive fusion; psychological rigidity | Treating the self as a fixed, describable object detached from lived engagement |
| Idem-identity (sameness) | Rigid self-concept; over-identification | The “what” of identity: stable predicates and categories that define the self |
| Ipseity (selfhood, *ipse*) | Self-as-context; flexible self-awareness | The “who” of identity: the relational, temporal mode of being that cannot be objectified |
| Constative language | Literalized self-talk; narrative self-reference | Language that describes or states facts about the self as fixed truths |
| Performative language | Defused or experiential speech | Language that enacts or constitutes a mode of relating rather than describing |
| Direct reference | Experiential awareness; felt sense | Meaning arising from lived experience without objectification |
| Indirect reference | Conceptual/representational self-reference | Meaning mediated through abstract categories that objectivize experience |
| Indeterminacy of the self | Psychological flexibility; openness to experience | The absence of a final, fixed ground for self-definition |
| *Angst* (existential anxiety) | Distress tolerance; affect regulation in transition | Anxiety arising from confrontation with the groundlessness of existence |
| Non-duality | Decentering; non-attachment to self-narratives | The experiential recognition that self and experience lack fixed, independent essence |
